# Honey vs. pharmacological treatment for acute cough in children: a systematic review and meta-analysis of randomized controlled trials

**DOI:** 10.1007/s00431-026-07348-w

**Published:** 2026-08-31

**Authors:** Mariana Timm Brun, Maria Clara Abreu Vianna Braga, Sabrine Teixeira Ferraz Grunewald

**Affiliations:** 1 Medical College, Sector of Health Sciences, Federal University of Paraná, 280 Padre Camargo St, Alto da Glória, Curitiba, Paraná 80060-240 Brazil; 2 Medical College, Salvador University, Salvador, Bahia Brazil; 3 Maternal and Child Department, Federal University of Juíz de Fora, Juíz de Fora, Minas Gerais Brazil

**Keywords:** Acute cough, Honey, Meta-analysis, Pediatrics, Pharmacologic, Systematic review

## Abstract

This systematic review and meta-analysis of randomized controlled trials (RCTs) aimed to compare honey with pharmacological treatment in pediatric patients with acute cough. Studies were searched across 5 databases. Analyzed outcomes were cough frequency, severity, and bothersomeness, alongside its impact on the sleep of both children and caregivers. A random-effects meta-analysis was performed using the *Review Manager Web* tool. A post-hoc subgroup and leave-one-out sensitivity analysis were conducted to evaluate the influence of individual studies and medication class on heterogeneity. 7 studies were included in the systematic review and 6 in the meta-analysis, totaling 576 patients. Among them, 305 (53%) received honey. Participants’ mean age ranged from 3 to 5 years, and follow-up duration was limited to 3 days. The primary analysis showed high heterogeneity and no statistically significant differences across most endpoints, except for cough effect on child's sleep, which was significantly reduced in the honey group (MD: -0.73; 95% CI: -1.45 to -0.01; p = 0.05). After sensitivity analysis, honey significantly improved all outcomes, including cough frequency, severity, bothersomeness, sleep quality of children and caregivers, and global score. Subgroup analyses confirmed consistent results regardless of the pharmacological comparator used. Alone, the honey-vs-dextromethorphan group showed significant benefit in two endpoints.

*Conclusion*: While the primary analysis demonstrated comparable efficacy between interventions across most endpoints, except for child sleep quality, exploratory post-hoc sensitivity analyses revealed a potential signal of honey superiority across all outcomes. Although these findings should be regarded as hypothesis-generating rather than definitive, the potential clinical benefit, combined with the established risks of pediatric antitussives, supports honey as a promising, low-risk alternative.

*Trial registration*: CRD420261356244.

**Table:** 

**What is Known:** • *Pediatric acute cough commonly leads to inappropriate antitussive medication prescribing.* • *Honey outperforms placebo or no treatment for cough symptoms relief ≥ 10 or comorbidities.* **What is New:** • *Post-hoc sensitivity analysis suggests that honey is more effective than pharmacological therapy across all symptom domains.* • *Honey should be actively promoted over antitussives medications in children, given its safety and low cost.*

**Supplementary Information:**

The online version contains supplementary material available at https://doi.org/10.1007/s00431-026-07348-w.

## Introduction

Acute cough is defined as a cough lasting less than four weeks [1, 2]. Especially when nocturnal, this pediatric symptom results from various acute respiratory tract infections and conditions, representing a frequent and clinically significant problem in pediatric practice, with considerable repercussions on the quality of life and well-being of both children and their caregivers [1–4]. Furthermore, this condition constitutes a recurrent reason for seeking medical attention, including in emergency department services [1, 2]. Thus, the administration of antitussive medications, which are frequently over-the-counter (OTC), constitutes a common practice among parents, although their indiscriminate use has been associated with substantial risks and the occurrence of serious adverse events in children [5–9].

Historical evidence demonstrates that honey has been utilized as a therapeutic agent for millennia, with human use traced to some 8,000 years ago as depicted by Stone Age cave paintings [10–12]. From Ancient Egypt, where the Ebers Papyrus described its use for wounds and respiratory complaints [10–12], to Traditional Chinese medicine texts from documenting its use [12], honey has been consistently employed across cultures. In the medieval period, Avicenna further systematized its medicinal use, recommending it specifically for lung conditions and wound care [12]. Its widespread documentation across sacred and cultural texts throughout history proves its enduring significance in human healthcare [10–12]. Honey is also broadly categorized by its floral origin into monofloral varieties, derived primarily from a single plant species, such as *buckwheat, thyme, astragalus, eucalyptus, or citrus*, and polyfloral formulations [13]. These varieties differ substantially in color, sugar ratios, phenolic content, and antioxidant capacity, with darker honeys generally displaying higher concentrations of bioactive compounds [13].

Nowadays, honey has emerged as a promising and low-cost therapeutic alternative for the treatment of childhood cough [14–23], especially in the current scenario, characterized by a growing search for non-pharmacological therapies and by the awareness regarding the deprescribing of unnecessary medications [24–26]. Previous systematic reviews have demonstrated the superiority of honey compared to placebo and no treatment in relieving these symptoms in childhood [15, 22]. However, its efficacy directly compared to the most common antitussives for acute conditions has not yet been fully clarified. Although isolated investigations have shown significant advantages of honey over dextromethorphan and other medications [14, 16–21], guidelines for the management of pediatric cough still recommend its use empirically [27, 28]. Consolidated data from meta-analyses exclusively comparing these interventions remain scarce in the literature, since previous reviews pooled control groups involving placebo and no-treatment rather than isolating the direct efficacy of honey against pharmacological antitussives [15, 22, 23].

Therefore, the objective of this study is to perform a systematic review and meta-analysis of RCTs comparing the relative efficacy of honey to antitussive medications in pediatric acute cough. The investigation focused on children over 1 year of age presenting with multiple etiologies.

## Material and methods

This systematic review and meta-analysis was conducted in accordance with the *Cochrane Collaboration Handbook for Systematic Reviews of Interventions* and reported following the *Preferred Reporting Items for Systematic Reviews and Meta-Analyses* (PRISMA) 2020 guidelines [29, 30].

### Eligibility criteria

The eligibility criteria for inclusion in this meta-analysis comprised: (a) being an RCT; (b) evaluating children over 1 year of age; (c) addressing acute cough; (d) comparing the use of honey with antitussive medications; and (e) presenting a reported follow-up of up to 3 days. Additionally, the studies had to report at least one of the outcomes of interest evaluated in this review. The minimum age criterion of 1 year was applied because honey is not recommended for younger infants due to the risk of infant botulism [1, 31, 32]. The restriction of follow-up to up to 3 days was adopted considering the spontaneous regression of cough over time. Non-randomized and placebo or no treatment exclusive assessments studies were excluded. There was no restriction on the language of studies.

### Search strategy and data extraction

A systematic search was conducted in the *Cochrane Central Register of Controlled Trials* (CENTRAL), EMBASE, PubMed (MEDLINE), Scopus, and *Web of Science* databases in April 2026, with the following keywords: “*honey”, “bee”, “cough”, “cold”, “respiratory infection”, “bronchiolitis”, “asthma”, “pneumonia”, and “children”*. Full search strategy can be found in the Supplementary Appendix. Additionally, a manual check (backward snowballing) of all included studies, reviews and meta-analyses references was performed. Data were independently extracted by two authors (MTB and MCAVB) using predefined eligibility criteria. Disagreements were subsequently reviewed and resolved by the senior author (STFG). The protocol for this meta-analysis was registered in PROSPERO (CRD420261356244). Dose, dosing frequency and treatment duration were extracted as additional variables for both arms (Supplementary Table 1).

### Outcomes

In this meta-analysis, primary outcomes evaluated included scores for cough frequency, severity, and bothersomeness; impact of cough on the sleep of both children and parents; and global score. The definitions of these scores were based on a 7-point Likert scale ranging from 0 (absence of symptoms) to 6 (extreme symptoms) previously proposed [14] (Supplementary Table 2). No secondary endpoints were evaluated. Although included in the systematic review, one study [21] was subsequently excluded from the meta-analysis due to incompatibility in the verification of endpoint data. The study by Sopo et al. reported only the combined symptom score and was therefore included exclusively in the global score analysis [20].

### Quality assessment

The risk of bias was investigated using the *Cochrane Risk of Bias* (RoB 2) tool [33] by two authors (MTB and MCAVB). Disagreements were subsequently reviewed and resolved by the senior author (STFG). The certainty of the evidence was assessed using the *Grading of Recommendations Assessment, Development and Evaluation* (GRADE) [34] approach by two authors (MTB and STFG).

### Statistical analysis

The continuous outcome was analyzed through mean difference (MD) with a 95% confidence interval (CI). In studies that reported results exclusively as median and interquartile range (IQR), mathematical conversion to mean and standard deviation (SD) was performed using the methods proposed by Luo et al. (2018) for mean estimation and Wan et al. (2014) for SD estimation [35, 36]. In multi-armed trials [14, 18–22], population data were combined in an aggregate manner for descriptive purposes in Table 1. For the subgroup analysis, each pharmacological comparator was analyzed separately, and the honey group sample size was split equally across subgroups while means and SDs were kept identical, following *Cochrane Handboo*k (Sect. 23.3) [29]. Study identifiers were suffixed with a letter to allow independent entry within each subgroup.

**Table 1:** Baseline characteristics of included studies

Study	Country	Control arms included, *n*	Honey group, *n*	Mean ± SD age, years	Female, *n* (%)	Mean ± SD cough frequency	Mean ± SD cough severity	Mean ± SD cough effect on child’s sleep	Mean ± SD cough effect in caregiver’s sleep	Follow-up
Anibasa, 2022	Nigeria	DPH 42	42	3.61 ± 1.99	37 (44%)	5.00 ± 1.23	4.00 ± 1.48	4.00 ± 1.48	4.00 ± 1.48	2 nights
Ayazi, 2017	Iran	DPH 20	67	3.50 ± 1.60	46 (53%)	3.51 ± 0.95	3.84 ± 0.82	3.05 ± 1.15	3.61 ± 0.96	2 nights
Paul, 2007	USA	DM 33	35	4.94 ± 2.85	34 (50%)	3.88 ± 1.02	3.97 ± 1.04	3.82 ± 1.17	4.00 ± 1.39	24 h
Shadkam, 2010	Iran	DM 36 DPH 34	33	3.14 ± 0.93	52 (51%)	4.13 ± 0.79	3.88 ± 0.83	3.64 ± 0.68	3.92 ± 0.70	24 h
Sopo, 2015	Italy	DM 29 LDP 34	71	NA	NA	NA	NA	NA	NA	3 nights
Waris, 2014	Kenya	SABA 43	57	NA	46 (46%)	3.19 ± 0.79	2.98 ± 1.05	2.60 ± 1.33	2.54 ± 1.05	3 days

*DM* Dextromethorphan, *DPH* Diphenhydramine, *LDP* Levodropropizine, *NA* Not available, *SABA* Salbutamol, *SD* Standard deviation, *USA* United States of America

When the numerical outcomes of interest were not expressed in text or tables [20], contact with authors was attempted; without response, data were graphically extracted using the open-source software *WebPlotDigitizer* [37]. For studies omitting the SDs [14, 18], following unsuccessful attempts to contact the authors, the missing values were imputed using the largest SD observed among other studies included, as recommended by the *Cochrane Handbook*.

Statistical analyses were performed in the *Review Manager Web* (RevMan) software [38]. Given the expected clinical heterogeneity across studies, quantitative results were calculated under a random-effects model, applying the *Restricted Maximum-Likelihood* (REML) method for Tau^2^ calculation and the *Hartung-Knapp-Sidik-Jonkman* (HKSJ) method to provide more robust CIs. Statistical heterogeneity was assessed by the I^2^ test, with values above 25% considered indicative of significant heterogeneity. Given the pharmacological heterogeneity of the comparator arm, which included agents with distinct mechanisms of action, a post-hoc subgroup analysis stratified by pharmacological class was performed to allow more clinically meaningful comparisons. Additionally, a post-hoc *leave-one-out* sensitivity analysis (sequential omission of each individual study) was performed to evaluate the impact of each trial on overall heterogeneity and ensure the robustness of the estimated results.

## Results

### Study selection and baseline characteristics

**Fig. 1:**
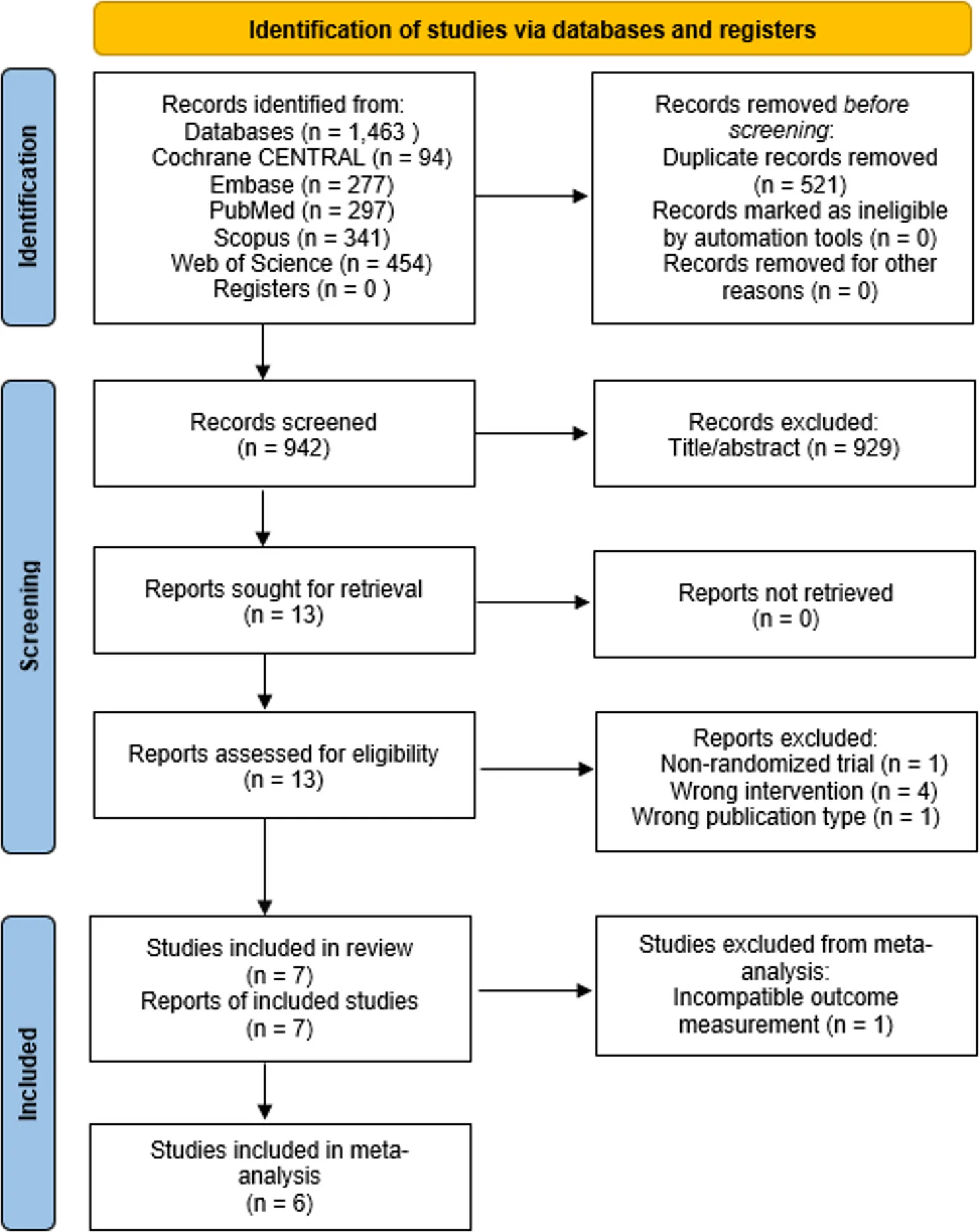
PRISMA 2020 flow diagram of study selection process

The initial search retrieved a total of 1,463 records. After removing duplicates and screening titles and abstracts, 13 studies were selected for full-text reading. Of these, 7 RCTs met all eligibility criteria and were included in the systematic review. For the meta-analysis, 6 studies were included, and 1 study was excluded due to incompatibility in endpoint analysis, totaling 576 patients (Fig. 1). Reasons for exclusion are justified in the Supplementary Appendix. A detailed description of the included and excluded arms for each study is provided in the Supplementary Table 3.

Among the 576 analyzed patients, 305 (53%) received honey. Of those allocated to the control arms, 96 (17%) received diphenhydramine, 98 (17%) received dextromethorphan, 34 (6%) received levodropropizine, and 43 (7%) received salbutamol. Baseline characteristics of the study population are summarized in Table 1. Dosing regimens varied across trials (Supplementary Table 1). Honey was administered at 2.5 to 10 mL per dose, adjusted by age band in four trials and given as a fixed dose in the remaining two. Comparator doses ranged from 7.5 to 30 mg for dextromethorphan, 6.25 to 12.5 mg for diphenhydramine, and 1 to 3 mg for salbutamol; levodropropizine was dosed at 1 drop/kg (Supplementary Table 1).

### Pooled analysis of all studies

**Fig. 2:**
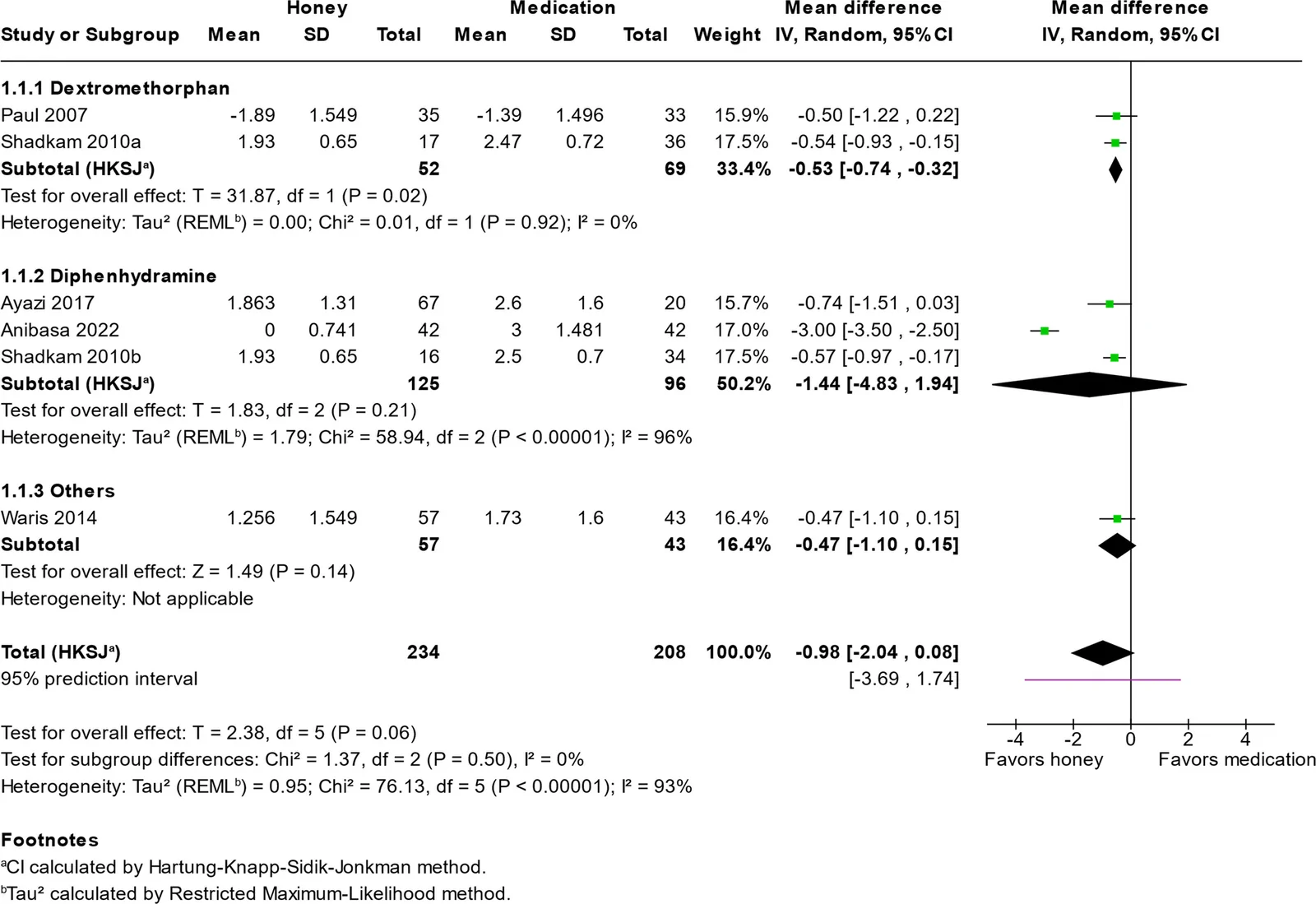
Forest plot for cough frequency shows no difference between groups

No statistically significant difference was observed between two groups in the score for cough frequency (MD: −0.98; 95% CI: −2.04 to 0.08; p = 0.06; I^2^ = 93%; Fig. 2), cough severity (MD: −1.00; 95% CI: −2.09 to 0.09; p = 0.06; I^2^ = 96%; Supplementary Fig. 1), or cough bothersomeness (MD: −0.60; 95% CI: −1.21 to 0.01; p = 0.05; I^2^ = 59%; Supplementary Fig. 2).

**Fig. 3:**
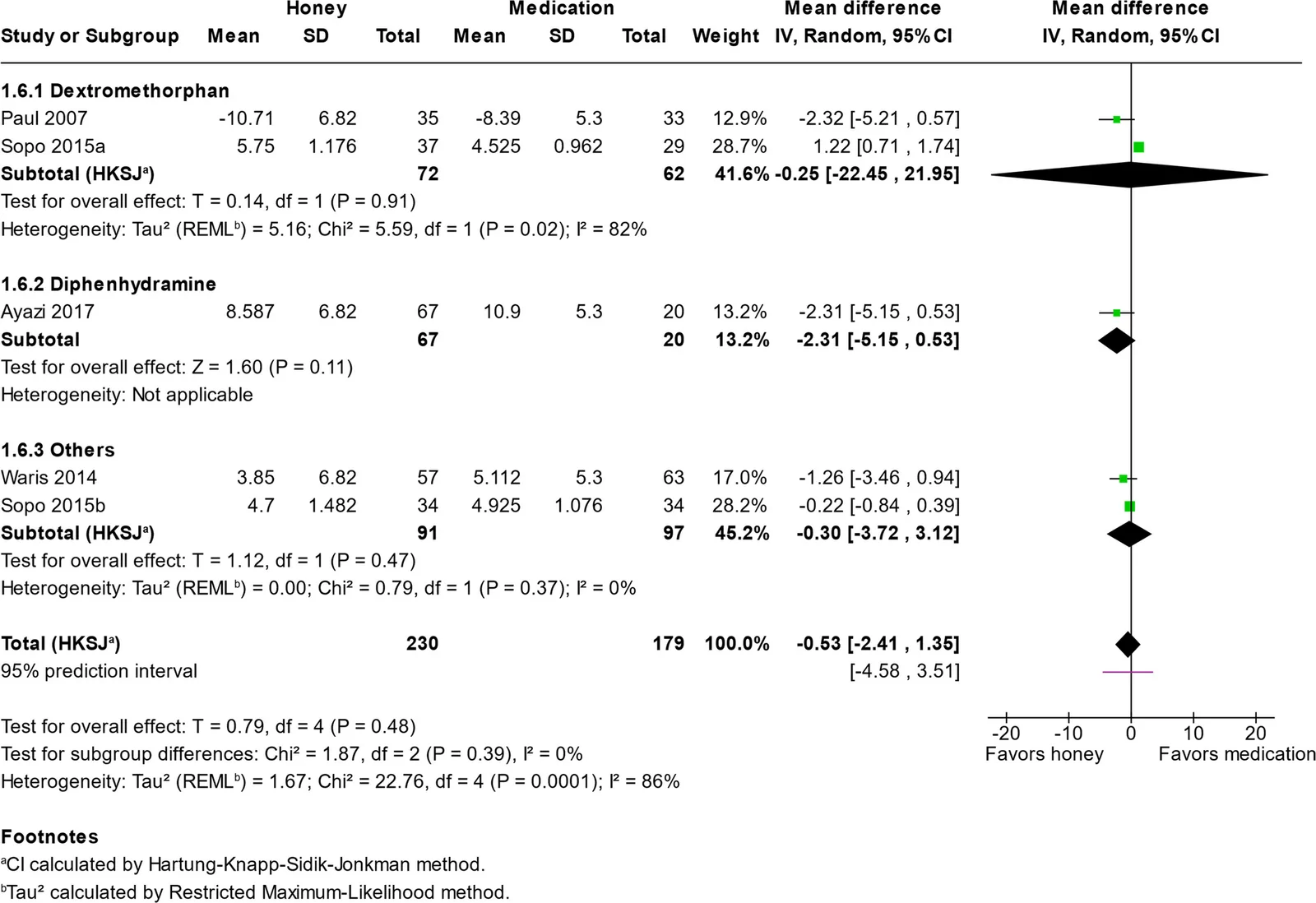
Forest plot for global score shows no difference between groups

**Fig. 4:**
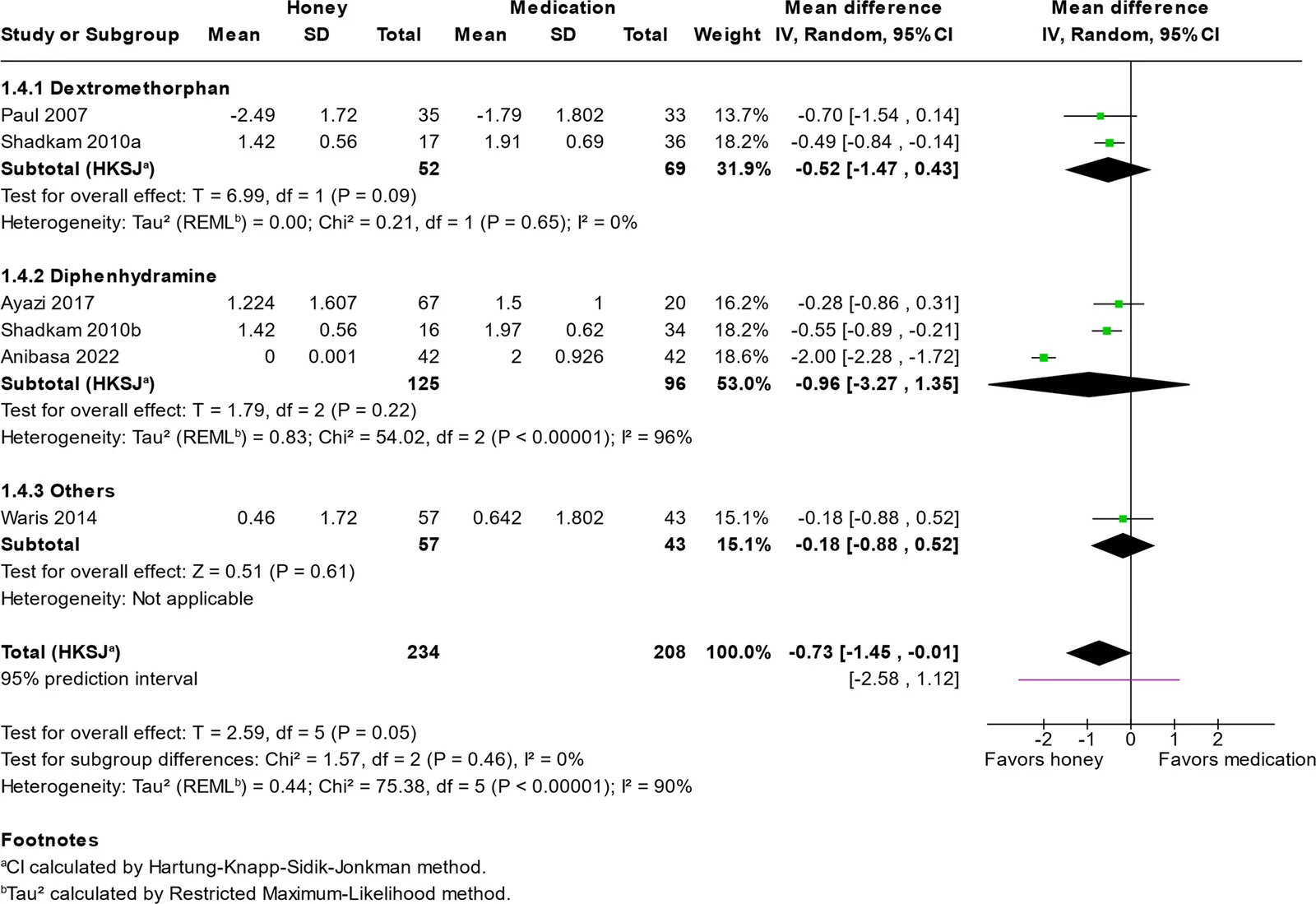
Forest plot for cough effect on child’s sleep shows significant benefit in the honey group

Additionally, there was no statistically significant difference between honey and medications for cough effect on caregiver’s sleep (MD: −0.60; 95% CI: −1.39 to 0.20; p = 0.11; I^2^ = 92%; Supplementary Fig. 3) and global score sum (MD: −0.53; 95% CI: −2.41 to 1.35; p = 0.48; I^2^ = 86%; Fig. 3). Nonetheless, a statistically significant improvement in child’s sleep was observed in the honey group compared to the pharmacological group (MD: −0.73; 95% CI: −1.45 to −0.01; p = 0.05; I^2^ = 90%; Fig. 4).

### Subgroup analysis

Low between-subgroup heterogeneity was demonstrated across most outcomes. No significant differences were detected between subgroups for cough frequency (p = 0.50; I^2^ = 0%), cough bothersomeness (p = 0.37; I^2^ = 0%), child sleep quality (p = 0.46; I^2^ = 0%), caregiver sleep quality (p = 0.49; I^2^ = 0%), or global score (p = 0.39; I^2^ = 0%). For cough severity, a degree of heterogeneity was observed (p = 0.24; I^2^ = 30.4%).

Regarding the comparison between honey and dextromethorphan, honey demonstrated superiority in alleviating cough frequency (MD: −0.53; 95% CI: −0.74 to –0.32; p = 0.02; I^2^ = 0%) and reducing its impact on caregiver sleep (MD: −0.39; 95% CI: −0.63 to –0.16; p = 0.03; I^2^ = 0%). Other subgroups and outcomes did not reach relevant significant differences between interventions.

### Sensitivity analysis

Due to the identified heterogeneity, a *leave-one-out* sensitivity analysis was performed for all outcomes. This analysis demonstrated that the study by Anibasa 2022 [16] was the primary driver of both statistical heterogeneity and observed results. Complete findings are available in the Supplementary Appendix.

Following the exclusion, honey demonstrated statistically significant superiority in reducing cough frequency score (MD: −0.55; 95% CI: −0.65 to −0.46; p < 0.0001; I^2^ = 0%; Supplementary Fig. 4) and cough severity score (MD: −0.60; 95% CI: −0.84 to −0.37; p = 0.002; I^2^ = 0%; Supplementary Fig. 5). Similarly, the outcomes for cough bothersomeness (MD: −0.35; 95% CI: −0.63 to −0.06; p = 0.04; I^2^ = 0%; Supplementary Fig. 6) became significantly favorable to the honey group. Notably, the removal of this study [16] reduced statistical heterogeneity across all these outcomes.

The beneficial effects of honey on child’s sleep quality remained significant (MD: −0.47; 95% CI: −0.66 to −0.28; p = 0.002; I^2^ = 0%; Supplementary Fig. 7), and statistical heterogeneity decreased. As for the impact of cough on caregiver sleep, the exclusion attenuated its heterogeneity and favored the honey group (MD: −0.35; 95% CI: −0.60 to –0.09; p = 0.02; I^2^ = 0%; Supplementary Fig. 8). Similarly, in combined symptoms, the removal of the trial by Sopo 2015 [20] reduced heterogeneity and significantly benefited the honey group (MD: −1.83; 95% CI: −3.43 to −0.23; p = 0.04; I^2^ = 0%; Supplementary Fig. 9).

### Quality assessment

One study was considered to have a high risk of bias, while all others showed some concerns. Detailed information regarding this assessment is available in Supplementary Table 4. The results concerning the certainty of the evidence by GRADE will be found in Supplementary Table 5.

## Discussion

This systematic review and meta-analysis, encompassing 6 RCTs and a total of 576 pediatric patients, evaluated the efficacy of honey compared to conventional antitussive medications. In the initial analysis, no statistically significant differences were observed between groups for cough frequency, severity, and bothersomeness, nor for the impact of cough on the sleep quality of parents, and global scores combination. The child’s sleep quality, however, was significantly benefited from the use of honey. Upon conducting the sensitivity analysis and excluding heterogeneous studies, the honey-treated group demonstrated statistically significant superiority, showing a reduction in cough frequency, severity, bothersomeness and global scores, in addition to an improvement on children's and parents’ sleep quality. Subgroup analyses further indicated consistent honey efficacy across all pharmacological regimens.

The lack of initial divergence between honey and conventional drugs stems primarily from the natural history of acute pediatric cough, which arises predominantly from benign, self-limiting viral infections [39]. As demonstrated, the natural course of this symptom in children tends toward spontaneous resolution even in the absence of any intervention [14, 40], justifying the authors’ decision to limit the follow-up period to up to 3 days.

The signal of superiority suggested by the post-hoc sensitivity analysis is biologically plausible and may be supported by honey’s synergistic properties. Honey acts as a demulcent agent in the pharynx, mechanically modulating local receptors by forming a protective film that attenuates tussigenic irritation [2]. The presence of compounds with anti-inflammatory and antimicrobial properties directly contributes to reducing upper airway edema, accelerating symptomatic relief [41, 42]. Additionally, its characteristic sweetness plays a neurophysiological role: it reflexively stimulates salivation and mucus secretion, promoting airway lubrication, while simultaneously activating oral sensory fibers that interact with the brainstem, generating a competition of stimuli capable of dampening the neurological cough reflex [14, 43].

Attention should be directed to the selective benefit on children's sleep quality, an outcome that emerged prior to sensitivity analysis. While high heterogeneity necessitates a cautious interpretation, this result suggests potential neurophysiological mechanisms through which honey modulates sleep pathways. Specifically, honey harbors serotonin, melatonin, and their functional precursors in quantifiable amounts [44, 45]. Additionally, the carbohydrates present in honey may enhance central tryptophan transport across the blood–brain barrier by outcompeting large neutral amino acids, ultimately driving endogenous melatonin synthesis [46]. Together with its demulcent capacity to soothe nocturnal pharyngeal irritation [2], these combined physiological properties account for honey’s selective superiority in this specific clinical domain.

Although prior meta-analyses have validated the efficacy of honey in managing upper respiratory tract infections [15, 22, 23], their scopes included both adult populations and control groups based on placebo or no treatment. In contrast, this study focuses exclusively on the pediatric population and directly compares honey with conventional antitussive medications. By performing a sensitivity analysis that restored methodological homogeneity, this meta-analysis offers pediatricians and primary care physicians evidence of greater specificity. These findings provide statistical support for major international clinical guidelines, such as those from the *National Institute for Health and Care Excellence* (NICE) and the *American Academy of Pediatrics* (AAP) [27, 28], which already recommend honey for acute cough in children over one year of age.

Concurrently, the literature links the use of OTC antitussives to severe and potentially fatal adverse events in pediatric patients [5–7, 9]. Common drugs, such as dextromethorphan, diphenhydramine, and codeine, have been associated with severe neurological toxicity including deep sedation, respiratory depression, and seizures, and cardiovascular toxicity due to accidental overdose [47, 48]. This vulnerability has prompted international regulatory agencies, such as the *U.S. Food and Drug Administration* (FDA) and the *European Medicines Agency* (EMA), to issue restrictive warnings against the use of these substances in minors [49, 50]. Therefore, continuing to prescribe these medications for self-limiting conditions lacks clinical support, reinforcing the urgency for safe alternatives.

Several features of the available evidence base account for the substantial heterogeneity observed and limit the precision of the pooled estimates. First, neither the dose nor the dosing schedule was standardised: honey ranged from 2.5 to 10 mL per dose and treatment from a single night to five days, with one trial using three daily doses rather than a single nocturnal one [18] (Supplementary Table 1). Second, the comparator arm encompassed agents with distinct pharmacological targets, including salbutamol, a bronchodilator with no antitussive indication [18]. The subgroup analysis, stratified by pharmacological class, nevertheless, showed no significant between-subgroup differences for any outcome, suggesting that the direction of the honey effect was not driven by any single class. Third, honey varieties differed across trials and were unspecified in two of them; although the demulcent and sweet-taste mechanisms underlying cough suppression are shared across botanical origins, phenolic and antioxidant content varies substantially between monofloral and polyfloral honeys [13], and a variety-specific contribution cannot be excluded. One trial further administered honey diluted in warm milk [20], which reduces the viscosity on which the demulcent effect depends. Finally, every included trial relied exclusively on subjective, caregiver-reported Likert scores; none incorporated objective measures such as acoustic cough counts, overnight audio recording or actigraphy. Because honey and syrup preparations differ in taste and appearance, caregiver blinding was incomplete in most trials, so expectation effects on subjective ratings cannot be ruled out.

These considerations require further research: trials should standardize honey type and dose, pre-specify comparison against a single pharmacological class and pair caregiver-reported scores with objective cough measurement. Such refinements would sharpen the estimate of effect. Given the very low certainty of the current evidence, these findings should be regarded as preliminary rather than a basis for changing clinical practice at this time.

### Limitations

This study presents limitations. Statistical heterogeneity was noted across all outcomes, partly addressed by the leave-one-out sensitivity analysis; however, findings based on the exclusion of a single influential study should be interpreted as hypothesis-generating rather than conclusive. Mathematical conversion of medians and IQRs, graphical data extraction, and imputation of missing SD values may have introduced marginal imprecision. None of the included studies systematically reported adverse events or treatment adherence data, precluding formal safety comparisons. The lack of standardization in honey type and medication doses across studies represents additional sources of variability. Furthermore, the overall certainty of evidence across all evaluated outcomes was assessed as very low. Finally, although PROSPERO registration preceded data extraction and statistical analyses, it was completed after database searches were initiated.

## Conclusion

This systematic review and meta-analysis initially showed no statistically significant difference between honey and conventional antitussives in the management of acute pediatric cough and its impact on the sleep of caregivers. Its impact on the child's sleep, in turn, was significantly reduced in the honey group. After excluding the primary heterogeneity driver in the sensitivity analysis, honey demonstrated significant superiority in reducing all analyzed outcomes, although this post-hoc finding should be regarded as hypothesis-generating. Importantly, the lack of significant differences in the subgroup comparisons suggests that the relative efficacy of honey remained consistent, regardless of the specific control medication, while dextromethorphan group alone showed significant benefit in two endpoints. These results provide no indication that pharmacological treatment outperforms honey. While safety reporting across trials was limited (with no adverse events recorded), honey’s overall favorable safety profile, low cost, and the well-documented risks of pediatric OTC drugs support its consideration for acute cough in children over one year of age.

## Supplementary Information

Below is the link to the electronic supplementary material.

Supplementary file1 (DOCX 2095 KB)

## Data Availability

All data generated or analyzed are included in this published article and its supplementary information files.

## Abbreviations

AAP: American Academy of Pediatrics
CENTRAL: Cochrane Central Register of Controlled Trials
CI: Confidence interval
EMA: European Medicines Agency
FDA: Food and Drug Administration
GRADE: Grading of Recommendations, Assessment, Development and Evaluations
HKSJ: Hartung-Knapp-Sidik-Jonkman
IQR: Interquantile range
MD: Mean difference
NICE: National Institute for Health and Care Excellence
OTC: Over-the-counter
PRISMA: Preferred Reporting Items for Systematic Reviews and Meta-Analyses
RCT: Randomized controlled trial
REML: Restricted Maximum-Likelihood
RoB 2: Risk of Bias 2
SD: Standard deviation
