# Supplementary material for: Honey vs. pharmacological treatment for acute cough in children: a systematic review and meta-analysis of randomized controlled trials
Source: Eur J Pediatr. 2026 Aug 31;185(9):706. doi: 10.1007/s00431-026-07348-w (PMC13529870; doi:10.1007/s00431-026-07348-w)
Supplement: Supplementary file 1 [file 431_2026_7348_MOESM1_ESM.docx]

Honey vs. Pharmacological Treatment for

Acute Cough in Children: A Systematic Review and Meta-Analysis of Randomized Controlled Trials

Mariana Timm Brun^1^ | Maria Clara Abreu Vianna Braga^2^ | Sabrine Teixeira Ferraz Grunewald^3^, MD, PhD

**Journal:**

European Journal of Pediatrics

**Corresponding author:**

Mariana Timm Brun

Federal University of Paraná

[marianabrun@ufpr.br](mailto:marianabrun@ufpr.br)

**Content**

[Full search strategy	2](#_Toc806493485)

[Reasons for exclusion after full-text review	4](#_Toc1388104529)

[Reason for meta-analysis exclusion	6](#_Toc328406070)

[Supplementary Table 1	6](#_Toc1646752917)

[Supplementary Table 2	7](#_Toc1865070839)

[Supplementary Table 3	7](#_Toc30233976)

[Supplementary Table 4	8](#_Toc1741052822)

[Supplementary Table 5	8](#_Toc1768517595)

[Supplementary figures	9](#_Toc930471618)

## Full search strategy

- Cochrane CENTRAL:

(honey OR bee OR bees) AND (cough OR bronchitis OR cold OR “Respiratory Tract Infections” OR “respiratory infection” OR “respiratory infections” OR bronchiolitis OR asthma OR pneumonia) AND (child OR child* OR infant OR baby OR babies OR pediatric OR pediatrics OR babe OR babes)

- Embase:

(‘honey’:ab,ti OR ‘bee ‘:ab,ti) AND (cough:ab,ti OR bronchitis:ab,ti OR cold:ab,ti OR ‘Respiratory Tract Infections’:ab,ti OR 'Respiratory Infection’:ab,ti OR bronchiolitis:ab,ti OR asthma:ab,ti OR pneumonia:ab,ti) AND (child OR child* OR infant OR baby OR babies OR pediatric OR pediatrics)

- PubMed:

(honey OR bee OR bees) AND (cough OR bronchitis OR cold OR “Respiratory Tract Infections”[Mesh] OR “respiratory infection” OR “respiratory infections” OR bronchiolitis OR asthma OR pneumonia) AND (child OR child* OR infant OR baby OR babies OR pediatric OR pediatrics OR babe OR babes)

- Scopus:

(TITLE-ABS-KEY(honey) OR TITLE-ABS-KEY(bee) OR TITLE-ABS-KEY(bees)) AND (TITLE-ABS-KEY(cough) OR TITLE-ABS-KEY(bronchitis) OR TITLE-ABS-KEY(cold) OR TITLE-ABS-KEY(“Respiratory Tract Infections”) OR TITLE-ABS-KEY(“respiratory infection”) OR TITLE-ABS-KEY(bronchiolitis) OR TITLE-ABS-KEY(asthma) OR TITLE-ABS-KEY(pneumonia)) AND (TITLE-ABS-KEY(child*) OR TITLE-ABS-KEY(infant) OR TITLE-ABS-KEY(baby) OR TITLE-ABS-KEY(babies) OR TITLE-ABS-KEY(pediatric*))

- Web of Science:

(AB=(honey) OR AB=(bee)) AND (AB=(cough) OR AB=(bronchitis) OR AB=(cold) OR AB=(‘Respiratory Tract Infections’) OR AB=(‘Respiratory Infection’) OR AB=(bronchiolitis) OR AB=(asthma) OR AB=(pneumonia)) AND (AB=(child*) OR AB=(infant) OR AB=(baby) OR AB=(babies) OR AB=(pediatric*))

## Reasons for exclusion after full-text review

- Non-randomized trial:
- Warren MD, Pont SJ, Barkin SL, Feigenbaum KS, Allison MA, Schmiege SJ, Kempe A, Dempsey AF. The effect of honey on nocturnal cough and sleep quality for children and their parents. Arch Pediatr Adolesc Med. 2007 Dec;161(12):1149-53. doi: 10.1001/archpedi.161.12.1149. PMID: 18056559
- Wrong intervention:

- Della Volpe A, De Luca P, De Lucia A, Martines F, Piroli P, D'Ascanio L, Camaioni A, La Mantia I, Di Stadio A. Single-center-single-blinded clinical trial to evaluate the efficacy of a nutraceutical containing Boswellia serrata, bromelain, zinc, magnesium, honey, tyndallized Lactobacillus acidophilus and casei to fight upper respiratory tract infection and otitis media. Healthcare (Basel). 2022 Aug 13;10(8):1526. doi: 10.3390/healthcare10081526. PMID: 36011184; PMCID: PMC9408187.
- Cohen HA, Hoshen M, Gur S, Bahir A, Laks Y, Blau H. Efficacy and tolerability of a polysaccharide-resin-honey based cough syrup as compared to carbocysteine syrup for children with colds: a randomized, single-blinded, multicenter study. World J Pediatr. 2017 Feb;13(1):27-33. doi: 10.1007/s12519-016-0048-4. Epub 2016 Jul 15. PMID: 27457790.
- Reena, Kulkarni; Ramachandran SK. Randomized controlled trial on the effect of vrisha ghrita in childhood asthma management. Int J Res Ayurveda Pharm. 2013;4(1):78-83. doi: 10.7897/2277-4343.04130.
- Sadr S, Kaveh S, Choopani R, Bayat H, Mosaddegh M. Efficacy and safety of Iranian poly herbal formulation (compound honey syrup) in pediatric patients with mild to moderate asthma: a randomized clinical trial. Galen Med J. 2017;6(4):291-301. doi: 10.22086/gmj.v6i3.884.
- Wrong publication type:
- Gilbert, Greg. Single dose of honey effective for cough in kids. J Natl Med Assoc. 2008 Apr;100(4):459. doi: 10.1016/S0027-9684(15)31289-X.

## Reason for meta-analysis exclusion

- Incompatible outcome measurement:
- Ahmadi M, Moosavi SM, Zakeri S. Comparison of the effect of honey and diphenhydramine on cough alleviation in 2-5 year-old children with viral upper respiratory tract infection. J Gorgan Univ Med Sci. 2013;15(2):8-13

## Supplementary Table 1

**Table S1** Dosing regimens of honey and comparator medications in the included studies

| **Study** | **Honey, type and dose** | **Medication, type and dose** | **Doses per day** |
| --- | --- | --- | --- |
| **Anibasa, 2022** | NA, 5-10ml | DPH, NA (5-10ml) * | 1x/day (bedtime) |
| **Ayazi, 2017** | Kimia, 2.5-5ml  Shahde-Golha, 2.5-5ml | DPH, 6.25–12.5 mg | 1x/day (bedtime) |
| **Paul, 2007** | Buckwheat, 2.5-10ml | DM, 8.5–34 mg | 1x/day (bedtime) |
| **Shadkam, 2010** | Kafi-Abad, 2.5ml | DM, 7.5 mg  DPH, 6.25 mg | 1x/day (bedtime) |
| **Sopo, 2015** | Wildflower, 10ml | DM, 7.5–30 mg LDP, 1 drop/kg | 1x/day (bedtime) |
| **Waris, 2014** | NA, 2.5-7.5ml | SABA, 1–3 mg | 3x/day |

DM = dextromethorphan; DPH = diphenhydramine; LDP = levodropropizine; NA = not available; SABA = salbutamol.

*For Anibasa 2022, the volume administered was matched to that of honey, but the concentration of the diphenhydramine syrup was not reported.

## Supplementary Table 2

**Table S2** Likert-type scoring for pediatric cough outcomes evaluation

|  | Cough frequency | Cough severity | Cough bothersomeness | Cough effect on child’s sleep | Cough effect on caregiver’s sleep |
| --- | --- | --- | --- | --- | --- |
| 0 | Not at all | Not at all | Not at all | Not at all | Not at all |
| 1 | Not much | Not much | Not much | Not much | Not much |
| 2 | A little | A little | A little | A little | A little |
| 3 | Somewhat | Somewhat | Somewhat | Somewhat | Somewhat |
| 4 | A lot | A lot | A lot | A lot | A lot |
| 5 | Very much | Very much | Very much | Very much | Very much |
| 6 | Extremely | Extremely | Extremely | Extremely | Extremely |

Note: This table provides a simplified representation of the questionnaire proposed in the study by Paul et al. and used in other studies.

## Supplementary Table 3

**Table S3** Included study arms and sample size calculation for meta-analysis

| Study | Study arms (n)* | Non-included study arms (n) | Analyzed sample, n |
| --- | --- | --- | --- |
| Anibasa, 2022 | 2 (84) | - | 84 |
| Ayazi, 2017 | 3 (87) | - | 87 |
| Paul, 2007 | 3 (105) | NT (37) | 68 |
| Shadkam, 2010 | 4 (139) | NT (36) | 103 |
| Sopo, 2015 | 3 (134) | - | 134 |
| Waris, 2014 | 3 (145) | PLA (45) | 100 |

NT = no treatment; PLA = placebo

* Number of study arms (total population sample size randomized) in the original clinical trial.

Note: Non-included study arms were excluded to ensure a direct head-to-head comparison between honey and active pharmacological antitussive therapies. For subgroup analysis, the honey arm sample size was split equally across pharmacological subgroups in multi-armed trials.

## Supplementary Table 4

**Table S4** Risk of bias summary for included studies (RoB 2)

| **Study** | **Bias from randomization process** | **Bias due to deviations from intended interventions** | **Bias due to missing outcome data** | **Bias in measurement of the outcomes** | **Bias in selection of the reported result** | **Overall risk of bias** |
| --- | --- | --- | --- | --- | --- | --- |
| **Anibasa 2022** | Low | Low | Low | Low | Some concerns | Some concerns |
| **Ayazi 2017** | Low | Some concerns | Low | Some concerns | Low | Some concerns |
| **Paul 2007** | Low | Some concerns | Low | Low | Low | Some concerns |
| **Shadkam 2010** | Low | Some concerns | Low | Low | Some concerns | Some concerns |
| **Sopo 2015** | Low | High | Low | Low | Some concerns | High |
| **Waris 2014** | Low | Some concerns | Low | Low | Some concerns | Some concerns |

Note: Low = low risk of bias; Some concerns = insufficient information reported in the original trial to assess potential bias; High = high risk of bias. Methodological quality was assessed according to the Cochrane Collaboration guidelines, as described in the main text.

## Supplementary Table 5

**Table S5** Summary of finding: honey versus medications for acute cough in children

| Outcomes | Medications | Honey | Number of participants  (studies) | Quality of the evidence (GRADE) |
| --- | --- | --- | --- | --- |
| Cough frequency (scale from 0 to 6) | The mean ranged across antitussives groups from 1.7 to 3.0 points | The mean in honey groups was 0.98 lower (-2.04 to 0.08) | 442  (5 studies) | ⊕⊝⊝⊝  VERY LOW  due to risk of bias, inconsistency and imprecision |
| Cough severity (scale from 0 to 6) | The mean ranged across antitussives groups from 1.3 to 3.0 points | The mean in honey groups was 1.00 lower (-2.09 to 0.09) | 442  (5 studies) | ⊕⊝⊝⊝  VERY LOW  due to risk of bias, inconsistency and imprecision |
| Cough bothersomeness (scale from 0 to 6) | The mean ranged across antitussives groups from 0.85 to 2.2 points | The mean in honey groups was 0.60 lower (-1,21 to 0.01) | 339  (4 studies) | ⊕⊝⊝⊝  VERY LOW  due to risk of bias, inconsistency and imprecision |
| Cough effect on child’s sleep (scale from 0 to 6) | The mean ranged across antitussives groups from 0.64 to 2.0 points | The mean in honey groups was 0.73 lower (-1.45 to -0.01) | 442  (5 studies) | ⊕⊝⊝⊝  VERY LOW  due to risk of bias, inconsistency and imprecision |
| Cough effect on caregiver’s sleep (scale from 0 to 6) | The mean ranged across antitussives groups from 0.65 to 2.0 points | The mean in honey groups was 0.60 lower (-1.39 to 0.20) | 442  (5 studies) | ⊕⊝⊝⊝  VERY LOW  due to risk of bias, inconsistency and imprecision |
| Combined symptoms (scale from 0 to 30) | The mean ranged across antitussives groups from 4.5 to 11.0 points | The mean in honey groups was 0.53 lower (-2.41 to 1.35) | 399  (4 studies) | ⊕⊝⊝⊝  VERY LOW  due to risk of bias, inconsistency and imprecision |

Note: Despite the high level of heterogeneity obtained, the authors chose not to downgrade the study due to inconsistencies in the outcomes, since the leave-one-out analysis showed no significant heterogeneity when the study by Anibasa et al. and/or Sopo et al. was removed.

## Supplementary figures

**Fig. S1** Forest plot for cough severity shows no difference between groups

**Fig. S2** Forest plot for cough bothersomeness shows no difference between groups

**Fig. S3** Forest plot for cough effect on caregiver’s sleep shows no difference between groups

**Fig. S4** Forest plot for cough frequency show significant benefit in the honey group after sensitivity analysis excluding studies driving heterogeneity

**Fig. S5** Forest plot for cough severity shows significant benefit in the honey group after sensitivity analysis excluding studies driving heterogeneity

**Fig. S6** Forest plot for cough bothersomeness shows significant benefit in the honey group after sensitivity analysis excluding studies driving heterogeneity

**Fig. S7** Forest plot for cough effect on child’s sleep shows significant benefit in the honey group after sensitivity analysis excluding studies driving heterogeneity

**Fig. S8** Forest plot for cough effect on caregiver’s sleep shows significant benefit in the honey group after sensitivity analysis excluding studies driving heterogeneity

**Fig. S9** Forest plot for global score shows significant benefit in the honey group after sensitivity analysis excluding studies driving heterogeneity
